# Deep sequencing revealed molecular signature of horizontal gene transfer of plant like transcripts in the mosquito *Anopheles culicifacies*: an evolutionary puzzle

**DOI:** 10.12688/f1000research.7534.1

**Published:** 2015-12-30

**Authors:** Punita Sharma, Tanwee Das De, Swati Sharma, Ashwani Kumar Mishra, Tina Thomas, Sonia Verma, Vandana Kumari, Suman Lata, Namita Singh, Neena Valecha, Kailash Chand Pandey, Rajnikant Dixit

**Affiliations:** 1 Host-Parasite Interaction Biology Group, National Institute of Malaria Research, Delhi, India; 2 Nano and Biotechnology Department, Guru Jambheshwar University, Haryana, India; 3 NxGenBio Lifesciences, Delhi, India

**Keywords:** Mosquito, feeding, Salivary gland, Plant like transcripts, microbial flora, malaria

## Abstract

In prokaryotes, horizontal gene transfer (HGT) has been regarded as an important evolutionary drive to acquire and retain beneficial genes for their survival in diverse ecologies. However, in eukaryotes, the functional role of HGTs remains questionable, although current genomic tools are providing increased evidence of acquisition of novel traits within non-mating metazoan species. Here, we provide another transcriptomic evidence for the acquisition of massive plant genes in the mosquito, *Anopheles culicifacies*. Our multiple experimental validations including genomic PCR, RT-PCR, real-time PCR, immuno-blotting and immuno-florescence microscopy, confirmed that plant like transcripts (PLTs) are of mosquito origin and may encode functional proteins. A comprehensive molecular analysis of the PLTs and ongoing metagenomic analysis of salivary microbiome provide initial clues that mosquitoes may have survival benefits through the acquisition of nuclear as well as chloroplast encoded plant genes. Our findings of PLTs further support the similar questionable observation of HGTs in other higher organisms, which is still a controversial and debatable issue in the community of evolutionists. We believe future understanding of the underlying mechanism of the feeding associated molecular responses may shed new insights in the functional role of PLTs in the mosquito.

## Introduction

Horizontal gene transfer (HGT), an evolutionary force that modulates the movement of genetic information between distantly related organisms, is well accepted in prokaryotes ^1– 3^. However, unlike prokaryotes, uncovering the functional role of HGT from eukaryotes to eukaryotes remains challenging ^4^. Nevertheless, the number of well-supported cases of HGT are rapidly increasing, but one fundamental question, whether observed HGT in the genome or transcriptome of higher eukaryotes is pseudogenic ^5– 7^ or plays any important role in the evolution in complex metazoans, still remains unclear ^8– 10^.

Currently, next-generation sequencing is emerging as an important tool to discover and understand the evolutionary relationship of the molecular codes identified from non-model organisms ^11– 15^. Recently, a series of good review articles have been published, where authors critically argued and discussed that HGT from symbiotic/free-living organisms ^1, 4, 16, 17^, can be an important mechanism to drive the acquisition of novel traits. However, documenting the role of HGTs may be more controversial for the massive gene transfer within non-mating interspecies of complex metazoans ^4, 18– 20^.

One of the most debatable and contradictory HGT being argued is the massive transfer of algal nuclear and chloroplast encoded genes to mollusks ^18, 19^. In fact, it has long been documented that the herbivore sea slug *Elysia chlorotica* carries a unique ability to harvest plastids (absence of nuclei) from its heterokont algal prey, *Vaucheria litorea*, and keeps plastids for several months in the digestive tract for long-term maintenance of photosynthesis and development. To explain this complex interaction, a possible hypothesis of the HGT of algal genes in the gut of the mollusk is under extensive investigation. Although, a series of recent transcriptomic analyses provide supportive evidences of massive HGTs, however the inability to find evidence of the algal associated nuclear genes in the egg genome further leaves an open question over HGT’s role in evolution ^18^.

Mosquitoes that transmit many deadly infectious diseases, e.g. malaria, dengue, chikunguniya etc. are emerging as a valuable model to understand multi-taxon interactions ^21^. For entomologists, unraveling the molecular and evolutionary complexity associated with dual feeding behavior in adult female mosquitoes, remains one of the unresolved central questions. Acquisition of nectar sugar by adult mosquitoes (in both sexes) is essential for regular metabolic energy, while blood meal by adult female mosquitoes is needed for egg production and life cycle maintenance ^22^. The evolution of blood feeding is believed to have arisen independently over 145–165 million years ago from herbivore insects ^23, 24^, which might have favored the evolution of the specialized feeding organ system such as proboscis ^25^, enlarged salivary glands ^26^, facilitating the fast acquisition of nutrient rich blood meal from vertebrate host.

For the last few years, we have investigated the salivary associated molecular factors that affect mosquito feeding behavior and *Plasmodium* transmission ^27, 28^. *Anopheles culicifacies* exists as a complex of at least five sibling species (A, B, C, D, E) with wide distribution ^29, 30^ and acts as an important rural malarial vector causing more than 65% of malaria cases in India. We believe that malaria transmission by *A. culicifacies* in rural areas could be attributed to its strong adaptation towards agricultural plain areas. However, there is no molecular explanation that exists in relation to feeding behavior, evolution and adaptation preference to the plain area. Therefore, to understand the complex biology and molecular genetics of this mosquito, recently we have initiated a series of multi-tissue transcriptomic studies. In our recent RNAseq analysis, we demonstrated for the first time that salivary glands are evolved with unique ability to meet and manage dual (sugar or blood) meal specific responses in the mosquito *A. culicifacies* ^31^. However, unexpectedly during functional annotation of the salivary RNAseq database we also observed a cluster of plant like transcripts (PLTs) for which nature of origin remains unclear. Here, in the present investigation, we aimed to predict and examine the molecular nature, origin and evolutionary relationship of these putative PLTs. Multiple validations by PCR, real time PCR, coupled with immunoblot analysis and immuno-florescence assay (IFA) provide strong evidence that PLTs are of mosquito origin and may encode active proteins for specific functions. Phylogenomic analysis predicts that adaptation to the nectar sugar uptake might have favored the acquisition of PLTs, possibly a unique case of HGTs in the mosquito. To the contrary over HGTs role in evolution, our investigation provides another evidence of the massive transfer of genes from plant to mosquito *A. culicifacies*. A comprehensive molecular analysis of the PLTs and ongoing metagenomic analysis of tissue associated microbiome provide initial evidence of how the mosquito evolved and adapted for feeding over plant host. To the best of our knowledge this is the first study defining the unique relationship of mosquito-plant-microbe interactions.

## Results & discussion

In an attempt to clarify the molecular complexity associated with dual feeding behavior evolution in the mosquito, currently we are focused on sequencing, generating and annotating large scale transcriptomic databases of the mosquito feeding machinery components, e.g. salivary glands, midgut, olfactory tissues etc. In fact mosquito salivary glands are bi-lobed single epithelial layered organs that initiate biochemical communication to the plant or vertebrate hosts. Over the last decade, several investigations in adult female mosquitoes have been valuable in identifying salivary specific molecular factors that facilitate fast blood meal uptake from a vertebrate host ^32^. But how salivary glands manage dual meal (sugar vs. blood) specific molecular responses remains unclear. Our recent RNAseq based comparative salivary transcriptomic analysis demonstrated that adult female mosquito salivary glands are evolved with a unique ability to manage and facilitate meal specific responses ^31^.

### Pilot discovery of plant like transcripts

**Figure 1.**
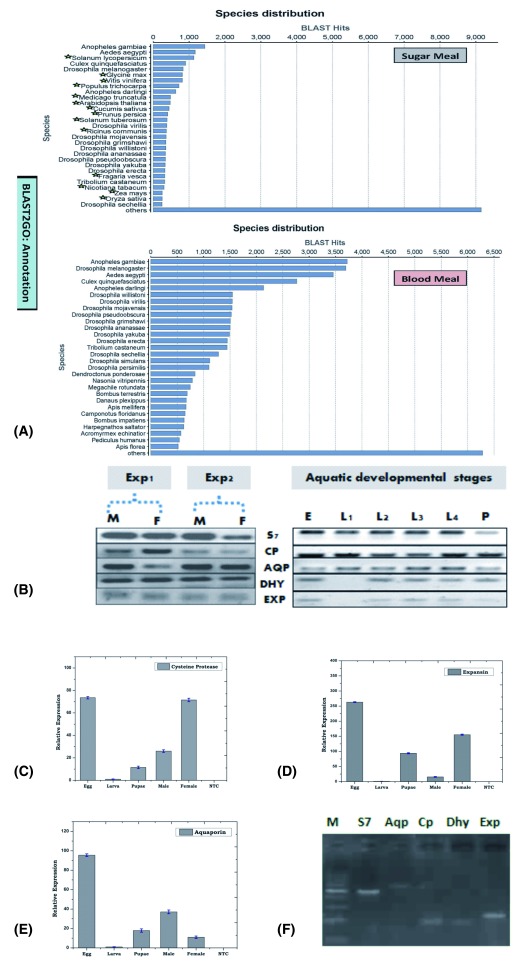
Mosquito encodes Plant like proteins. ( **A**) BLAST2GO based Species distribution analysis of sugar and blood fed mosquito salivary transcriptomic database. Green star mark indicates the name of plant species, best match to the NR database in the sugar fed salivary transcriptome. Confirmation of the nature of Origin ( **B**): RT-PCR expression of PLTs during aquatic development of the mosquitoes; ( **C**– **E**): Real-Time PCR based developmental expression analysis of PLTs viz. Cysteine protease; Expansin; Aquaporin; ( **F**): PCR based genomic DNA amplification of PLTs; **S7**: Ribosomal Protein S7; **Aqp**: Aquaporin; **Cp**: Cysteine protease; **Dhyd/Dhy**: Dehydrin; **Exp**: Expansin [(b): Exp1/Exp2: Experiment 1 & 2; M: Male; F: Female; E: Egg; L1–L4: Larval stages L1–L4; P: Pupae; (c) M: 100 bp Marker; NTC: No Template Control].

Interestingly, but unexpectedly our study ^31^ also revealed the presence of 537 putative transcripts encoding plant like proteins associated with the sugar fed library, but absent in the blood fed salivary transcriptome database ( Figure 1A; Supplementary material ST1). The surprising discovery of these transcripts, which we labeled as plant like transcripts (PLTs), raised several puzzling, but arguable questions that prompted us to clarify: (i) whether the PLTs are of mosquito origin; (ii) if they are expressed in the mosquito tissues and/or other developmental stages (iii) if expressed in mosquito, what is the possible evolutionary and functional correlation of these transcripts in feeding and (iv) whether these transcripts have any molecular relationship to plant-mosquito-microbe interactions/symbiotic associations. To uncover the molecular nature and possible functions of the putative PLTs, in the present investigation we performed a systematic and comprehensive analysis of PLTs, revealing a unique case of the massive transfer of HGTs from plant to mosquito.

### PLTs are of mosquito origin

**Figure 2.**
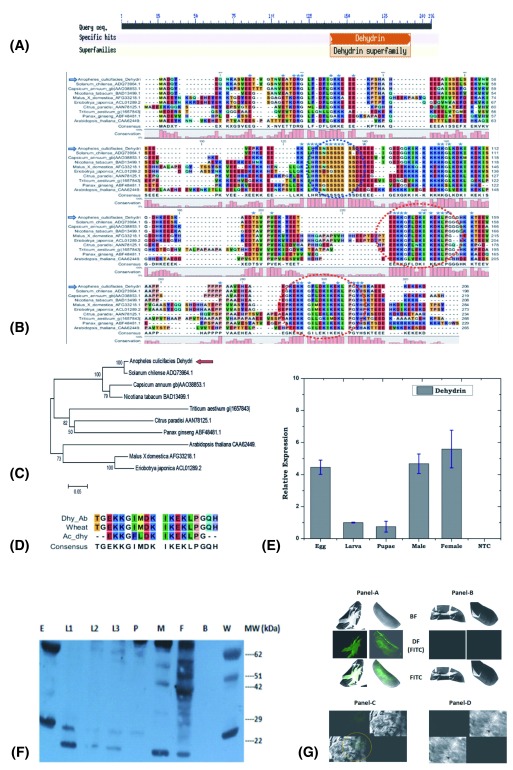
Functional validation of mosquito encoded Plant homolog Dehydrin. ( **A**) Web based functional prediction of putative domain of salivary transcript encoding plant homolog dehydrin like protein; ( **B**– **C**) Molecular and phylogenetic relation of mosquito encoded (Ac-Dehydrin with other plant dehydrins (dotted circle represents the conserved K/red circle and S/blue circle segments), a unique feature of plant dehydrins (see text); ( **D**) Mosquito dehydrin alignment of K-segment sequence with wheat and synthetic Dehydrin sequence used for antibody generation; ( **E**) Real-Time PCR based developmental expression analysis of *AcDehydrin*, ( **F**) Immunoblot analysis of Ac-Dehydrin expression during the development of the mosquito: Anti-dehydrin antibody recognize three protein bands of expected size (28, 52 & 63 kDa) in the control wheat seedling samples (W). Mosquito samples included Egg, Larval stages (L1, L2, L3), Pupa (P), Male (M) and Female (F); In egg stage two major forms of dehydrin bands were seen (≥62 kDa and 26 kDa). Further in case of larva, pupa, female, the presence of 26 kDa and 22 kDa bands suggested that two isoforms of dehydrin were present. In male, it appeared that less expression of 26 kDa form of dehydrin was seen in the western blot. Lane B represents Negative reference includes bacterial protein sample. The calculated molecular weight of 206 AA long salivary protein Ac-dehydrin is 23 kDa, which was closely similar in the larval stages. In fact dehydrins are characterized by conserved K-segment comprising consensus KIKEKLPG sequence towards the C-terminus and may be repeated one to many times, encoding variable size proteins ranging from 9 -200 kDa (see text for detail); ( **G**) Immuno-florescence assay: IFA analysis of Plant anti-dehydrin antibody binding and detection in the mosquito egg **Panel-A & B** and pupa **Panel-C & D.** The FITC labeled samples were observed and captured under bright field (BF), dark field (DF+FITC) and with FITC florescence signal only. Green florescence image (Panel-A or Panel-C) shows the presence of dehydrin expression in the mosquito. A corresponding image (Panel-B or Panel-D) of the samples represents the negative control samples processed under identical conditions, excluding primary anti-dehydrin antibody treatment only (see methods).

First, to confirm the nature of the PLTs’ origin, we did a deep enquiry with technical staff and confirmed that under standard rearing facilities, mosquitoes are never exposed to any plant material. To further rule out the possibilities of any contamination, we separately maintained the experimental mosquitoes as detailed in the methodology section. For technical validation of the PLTs’ origin, we conducted a series of experiments: (i) in two independent experiments, we examined and verified the RT-PCR based expression of at least 10 selected PLTs ( Figure 1B; Supplementary material S1A), in the salivary glands of adult male and female mosquitoes; (ii) interestingly, we also observed that PLT expression is not only restricted to the mosquito tissues, but is also expressed during the aquatic developmental stages viz. egg, larva, and pupa of the laboratory reared mosquitoes ( Figure 1B). Our relative gene expression analysis revealed that PLTs are more dominantly expressed in the egg, pupa and adult than larval stages ( Figure 1C–E). Although, mosquito egg and pupa stages are metabolically active, and do not take any food material, we suspected that the filter paper being used for mosquito egg laying may be a potential source of environmental contamination carry over. To clarify this doubt, we collected a small piece of moistened filter paper in RNA isolation solution (Trizol) and re-examined PLT expression along with other developmental stages. Absence of any amplification even after 35 PCR cycles, in the filter paper cDNA sample showed no sign of contamination ( Supplementary material S1A); (iii) we also observed positive amplification of selected PLTs through genomic DNA PCR ( Figure 1F); (iv) we further carried out the functional validation of one of the plant homolog PLTs encoding dehydrin protein, by immunoblot analysis as well as immuno-florescence assay ( Figure 2A–G); (v) lastly, from ongoing annotation of another independent transcriptome sequence database originated from non-salivary tissue i.e. olfactory (OLF) tissue of adult female mosquito *A. culicifacies* (Das De T., Sharma P., Thomas T., Pandey KC., Dixit R. unpublished data), we were able to observe similar PLTs ( Supplementary material S1B); (v) finally to test whether PLT expression is associated with feeding machinery components, we monitored the relative expression of PLTs in four tissues that included salivary glands, midgut, olfactory tissue and hemocytes, collected from 3–4 days old naïve adult female mosquitoes, by real-time PCR ( Figure 5A). Interestingly, we not only observed that PLTs are dominantly expressed in the tissues associated with mosquito feeding machinery (olfactory tissue, salivary gland and midgut), but also noticed a significant down regulation in response to blood meal in the salivary glands ( Figure 5A,B), evidencing that the mosquito genome may code plant like proteins.

### Mosquito encoded plant-homolog dehydrin: a functional validation

Dehydrins are a group 2 member of late embryogenesis abundant (LEA) proteins, originally identified from land plants, and known to be associated with desiccation (water stress) tolerance ^33^. In fact LEA proteins were thought to be restricted to plants and other lower eukaryotes viz. cyanobacteria, algae, but now they have also been identified in other animals including insects ^34^. Dehydrins are evolutionarily conserved proteins acclimated to low-temperatures (LT) that allow efficient tolerance to drought and cold stress among photosynthetic as well as some non-photosynthetic organisms such as yeast ^35– 38^. Dehydrins are characterized by lysine rich conserved K-segment comprising consensus amino acid sequence EKKGIMDKIKEKLPG, towards the C-terminus that may be repeated many times to encode 9 -200 kDa protein ^39– 42^. This unique feature renders these proteins cationic, providing cryoprotective activity towards freezing sensitive enzymes ^43^. The biochemical characterization of a novel cryoprotective protein in freeze-tolerant *Eurosta solidaginis* larvae shows dehyrin like activities ^44^, but a true homolog of dehydrin is yet to be verified.

Mosquito dehydrins have not been reported so far, though a putative transcript AGAP000328 has been predicted from the mosquito *A. gambiae* genome, carrying (PF00257 domain) a signature of dehydrin like proteins ( Supplementary material S2). Finding PLT encoding proteins associated with dehydration stress e.g. dehydrin, aquaporin, expansin etc. encouraged us to further examine their possible functions in the mosquito *A. culicifacies*. A comprehensive molecular analysis of the identified transcript *AcDehydrin* showed 100% identity to the plant dehydrin, having two conserved lysine rich K-segments ( Figure 2A–D). In our relative gene expression analysis, we observed a constitutive expression of *AcDehydrin*, throughout the aquatic developmental as well as adult stages of the mosquito ( Figure 2E), indicating that *AcDehydrin* transcript may encode a putative functional protein.

For functional validation of AcDehydrin protein, we examined the developmental expression of the dehydrin protein through immuno-blotting assay using rabbit antiserum containing anti-dehydrin antibody, raised against conserved K-segment sequence TGEKKGIMDKIKEKLPGQH ( Figure 2D) of plant dehydrin ^40^ (kind gift from Dr. Timothy Close). In our experiments we used wheat seedling protein sample as positive reference control. The anti-dehydrin antibody not only recognized the expected (28, 53 and 62 kDa) protein band in the wheat samples ^45^, but also identified at least two equivalent proteins (28 and 62kDa) abundantly expressing in different mosquito developmental stages viz. egg, adult male and female mosquitoes ( Figure 2F). Additionally, we were also able to observe multiple isoforms ranging from (~10 ->70 kDa) expressing at low level in different developmental stages, an expected unique feature of dehydrin to form macromolecular structures ^39– 42^. Finally, immuno-florescence assay not only corroborated the abundant expression in the egg, but also suggested that mosquito encoded AcDehydrin protein may play a crucial role in the stress tolerance and survival of the embryo in the egg ( Figure 2G).

Like other LEAs, dehydrins accumulate to high amounts in plant embryos, but remain undetectable in other vegetative tissues until their exposure to dehydration stress. The stress exposure results in their rapid induction and binding to multiple proteins, probably through intramolecular hydrogen bonding to protect tissue damage from dehydration/cold stress ^46^. In fact, we also find another key transcript, encoding a putative protein named expansin, a member of plant cell wall-loosening proteins. These proteins are known to be involved in cell enlargement and developmental processes requiring cell-wall modification ^47^. Like dehydrin, a real-time PCR analysis of expansin also showed dominant expression in the egg, as compared to other developmental stages ( Supplementary material S2B). Taken together, we hypothesize that the mosquito *A. culicifacies* may have survival benefits of cold stress tolerance as well as developmental regulation, similar to plants. Future studies involving dsRNA mediated gene silencing may unravel molecular and functional relationship of the PLTs controlling feeding and adaptation phenotypes in the mosquito ^48, 49^.

### Phylogenomic analysis of plant like transcripts

Next, to understand the possible evolutionary relationship we performed an extensive phylogenomic analysis of a few selected transcripts. To do this, first we retrieved and analyzed all plant-homolog putative transcripts (537 PLTs), and performed an extensive BLASTX analysis against either the NR database or an insect specific database at NCBI ( http://blast.ncbi.nlm.nih.gov/), and further characterized three categories of transcript(s) (i) one transcript: encoding highly conserved alpha-tubulin (cytoskeleton associated protein), showing highest identity to plant and insect (>95% and 85–90%, respectively); (ii) two transcripts: encoding aquaporin (water channel membrane protein) ^50^ and active site of the cysteine protease (protein chewing enzyme) ^51^ showing highest identity to plant (>90%) and 40–52% identity to insect (iii) two transcripts: encoding dehydrin (cold stress response protein) ^46^ and expansin (plant cell wall loosening protein) ^52^ only matched to plants, but remained unmatched to any insect database ( Supplementary material S3 A–C).

**Figure 3.**
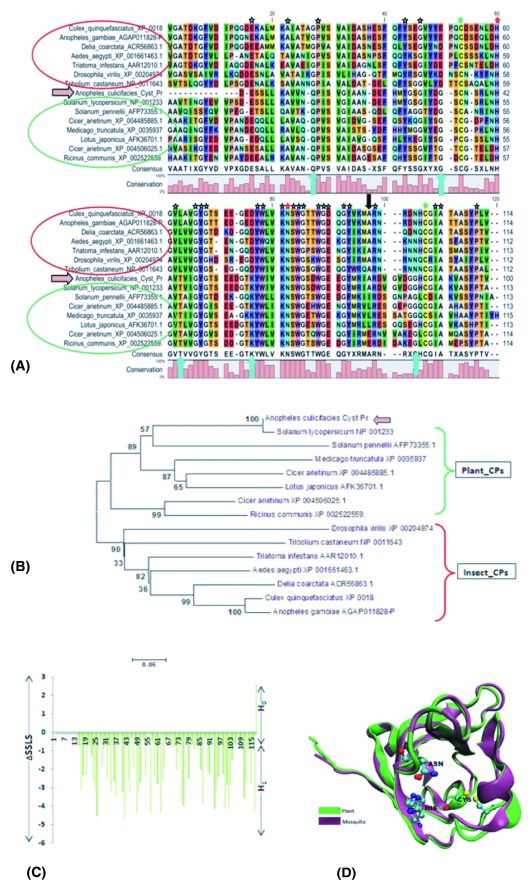
Feeding associated molecular complexity of the mosquito salivary transcripts. ( **A**) Molecular analysis of partial cDNA sequence encoding (100AA) Plant-like Cysteine protease active domain: Multiple sequence alignment showing molecular relationship of AcSgCp with plant (Green circle) as well as insects (Red circle) cysteine proteases: conserved residues (marked as 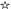) as well as conserved active site residue (marked as 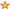). Green 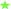 represents conserved cysteine residues, which enables disulfide formation. Upward arrow mark 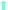 represent unique plant specific amino-acids residues also conserved in the *Anopheles culicifacies,* while downward arrow mark 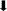 represent unique insect specific residue conserved in *A. culicifacies* and only in *Solanum lycopersicum*. ( **B**) The evolutionary history of AcSgCp inferred using the Neighbor-Joining method, favoring a clade formation with *S. lycopersicum* and other plant cysteine proteases. ( **C**) Relationship between strength of convergent evolution favoring adaptive significance of feeding associated PLTs: A maximum likelihood (ML) estimation was applied to calculate and compare the sitewise likelihood (∆SSLS) values between two, species evolution (H _0_) and convergent adaptive evolution (H _1_) hypothesis, for Cysteine protease (see text for details). ( **D**) Structural comparison between predicted 3D structure of the mosquito, and solved structure of the plants cysteine protease: Asparagine (ASN) and Histidine (HIS) indicate conserved residue of the active site.

The above results prompted to follow up the associated evolutionary consensus, favoring plant-mosquito relationship: a parallelism setting where different species from unrelated taxa faces the common selective pressure ^53^. Initial multiple sequence alignment analysis revealed significant heterogeneity (substitution/deletion) of amino acid residues, but also indicated unique conservation of insect or plant specific residues within the mosquito *A. culicifacies,* result a clade formation with plant species ( Figure 3A,B; Supplementary material S4). Subsequently, we also tested whether the evolution of common traits from unrelated taxa owing to similar selection pressure favors adaptive significance.

A maximum likelihood (ML) estimation was applied to calculate and compare the site-wise likelihood (∆SSLS) values between the two hypotheses, i.e. mosquito-mosquito species evolution (H _0_) and mosquito-plant convergent adaptive evolution (H _1_), for the selected PLTs. The site-wise log likelihood plot indicator, i.e. divergence towards negative (∆SSLS) was compared with LRT (likelihood ratio test), using the parametric bootstrap at 1000 replicate analysis (cut off p-value 5%). Final data analysis and comparison statistics favored the convergent hypothesis ^54^, demonstrating that mosquito *A. culicifacies* PLTs followed a convergent model favoring (H _1_), an adaptive evolution for sugar feeding associated functional relationship with plants ( Figure 3C; Supplementary material S4). Our analysis also supports the previous observations noted for the evolution of echolocating gene clusters among bats and bottlenose dolphins ^55^. Additionally, the predicted 3D structural analysis revealed fine conservation of the active functional domains in the mosquito and plant proteins e.g. cysteine protease ( Figure 3D; Supplementary material S5). From these studies, we concluded that mosquito feeding associated genes are not only evolving actively, but also acquiring new genes (e.g. dehydrin, expansin), to adapt successfully over the plant host.

### Feeding associated molecular complexity of ‘salivary-sugar-microbe’: A tripartite interaction

 Insect-plant association represents one of the most dominant interactions over millions of years ^56– 58^. These interactions are thought to play an important role in the co-evolution of molecular effector arms, enabling effective adaptation over each other ^59^. Uncovering of the molecular mechanisms of the herbivore insect-plant interaction has greatly facilitated the design of molecular strategies to save the valuable crops from insect pests ^60– 63^. However, such studies have not given special attention to mosquitoes. From the unexpected findings of the mosquito PLTs, we interpreted that either studies in relation to the sugar feeding associated biology have largely been ignored ^28^ or the mosquito *A. culicifacies* may have evolved with more complex genetic architecture favoring evolution of several environmentally-guided traits viz. carbon metabolism; light mediated photo conditions for mating, feeding, survival etc. Therefore, to predict sugar metabolism associated molecular and functional relationships of salivary PLTs, initially we analyzed all the putative PLTs against three databases (Reactome, KEGG, and Biocycles) annotated for *Arabidopsis thaliana*, using KOBAS online software, version 2.0 ( http://kobas.cbi.pku.edu.cn/home.do).

**Figure 4.**
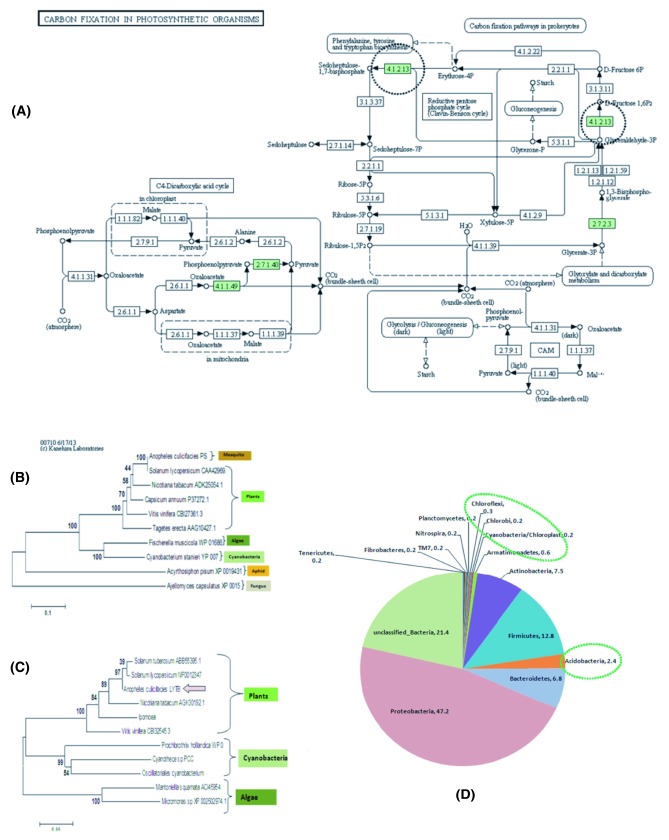
Molecular Evidence that mosquito encodes plant like photosynthetic machinery components partly shared by symbiotically associated salivary bacteria for carbon fixation and metabolism. ( **A**) KEGG prediction of salivary transcripts (differentially expressed/Fisher test p<0.001) encoding enzymes (Green) involved in “Carbon Fixation in Photosynthetic Organisms” pathway known to be restricted to the photosynthetic plants organelles e.g. plastids only (see text). ( **B**) Phylogenetic analysis of a unique mosquito salivary transcript, encoding a Plant homolog 4-hydroxy-3-methylbut-2-enyl diphosphate reductase/E.C.1.17.1.2 linked to the “Trepenoid Backbone Biosynthesis” pathway. ( **C**) Phylogenetic analysis of a unique mosquito salivary transcript, encoding a Plant homolog Phytoene Synthase/E.C.2.5.1.32 linked to the “Carotenoid Biosynthesis” pathway. In fact like other animals, insects are also believed to absorb carotenoid pigment (an eye pigment) from plant food. Additionally, lower microbes such as algae and cyanobacteria also carries LYTB/PS gene in their genome. Phylogenetic analysis of the salivary LYTB & PS showed unique association with the plant, as well as microbial LYTB while PS also showed evolutionary relationship to the novel PS gene recently identified from sap sucking insect *Acyrithosiphon pisum*, suggesting that mosquito LYTB/PS might have evolved, for independent synthesis of the carotenoid synthesis assisting feeding adaption preference over plant host. ( **D**) Identification of symbiotically associated salivary microbial flora predominated and unique bacteria (marked green circle), probably assisting mosquito to adapt, feed and metabolize diverse carbon rich sugar sources of plant origin (see another report for detail).

Notably, we observed that 18 transcripts encoding proteins related to at least five Biocyclic pathways linked to photosynthetic organelles viz. plastid in plants ( Supplementary material S6, T1). To verify the predicted ‘plastid’ related salivary transcripts, Fisher’s exact test was performed using BLAST2GO, revealing a pool of 11 transcripts differentially expressed in the sugar fed mosquitoes (Fisher test p<0.001; Supplementary material S6B) encoding important enzymes/proteins, associated with one of the key pathway “Carbon fixation in Photosynthetic Organisms” ( Figure 4A). Further, we also identified four unique salivary transcripts encoding different enzymes linked to three other secondary metabolite synthesis pathways, namely: ‘Trepenoid Backbone Biosynthesis’ (4-hydroxy-3-methylbut-2-enyl diphosphate reductase/E.C.1.17.1.2, LYTB); ‘Carotenoid Biosynthesis’ (Phytoene Synthase/E.C.2.5.1.32, PS); and ‘Flavonoid Biosynthesis’ (3-dioxigenase/E.C.1.14.11.9 & 3’ beta-hydroxylase/E.C.1.14.13.88) pathways restricted to plants ( Supplementary material S7). A comprehensive molecular and phylogenetic analysis of a few selected transcripts, encoding an enzyme 4-hydroxy-3-methylbut-2-enyl diphosphate reductase/E.C.1.17.1.2 (LYTB) and phytoene synthase/E.C.2.5.1.32 (PS) exclusively revealed a unique evolutionary relationship to the cyanobacteria, algae, plants and aphid *Acyrithosiphon pisum* ( Figure 4B,C; Supplementary material S7). In fact, during its early development mosquito larvae start to feed on diverse micronutrients e.g. bacteria, algae, fungi etc., and switch to feed on nectar sugars in adult mosquito stage. Thus, it could be possible that a long association and regular microbe-mosquito-plant interactions ^64, 65^, might have favored insects (mosquitoes) to adapt, feed, and digest sugar as well as selective synthesis of secondary metabolites/pigments, essential for specific phenotype e.g. visual pigmentation/dark body coloration ^66^. A recent study on light-induced ATP synthesis from the chloroplastid-like carotene pigments in *Acyrithosiphon pisum*, a plant sap sucking aphid, provides the first molecular evidence that the aphid genome may carry plant like photosynthetic machinery components ^67^. A fungal mediated lateral HGT mechanism has been proposed for the evolution of the carotenoid biosynthesis gene in this aphid ^20^.

In nature, mosquitoes are regularly exposed to various environmental factors which have adverse effects on their reproductive success, longevity & vector competence ^68^. Gut bacterial endosymbionts are known to play a part in several functions including food digestion, metabolism, reproduction and immunity ^69^. Our recent metagenomic analysis of salivary microbiome identified several unique bacterial phyla, including *Chlorobium, Cyanobacteria, Nitrospira* and other phototrophic bacteria associated with salivary glands ( Figure 4D), but absent in the gut of laboratory reared 3–4 days old adult female mosquitoes *A. culicifacies* ^70^. Indirectly, the above findings further support the hypothesis that mosquitoes may have feeding associated distinct plant like molecular machinery components, partly shared by the residing symbiotic bacterial community for diverse carbon/nitrogen rich plant sugar source metabolism. For example, finding of prominent salivary associated *Acidobacteria* (2.4%), may facilitate the utilization of plant polymer viz. cellulose/xylan sugars of diverse origin ^71^, as reported in the gut of the wood feeding larvae of Huhu Beetle ( *Prionoplus reticulari*) ^72^.

### HGTs in eukaryotes: a key to success

The observation of a large pool of chloroplast and nuclear encoded plant genes in the mosquito transcriptome supported the previous finding of similar gene transfer of photosynthetic machinery components from algae to mollusk ^19^. In addition to this, molecular analysis of PLTs also revealed a plant related class of secondary metabolites (see above) and immune genes i.e. Remorin (anti-fungal); osmotin/thaumatin (anti-fungal); and Vicilin (Antimicrobial) ( Supplementary material ST-1), a similar finding of active genes in the aphid genome ^73^. Although, in case of algal-mollusk or Aphid-plant interaction studies, the role of microbes is yet to be established, however, our metagenomic analysis provides initial evidence that tissue associated microbial flora may also share and facilitate optimal function. Thus, we believe that the accumulating data of genetic material transfer within metazoans are still at a premature stage, but emerging evidences strongly suggest that acquisition and retention of desired active functional genes for beneficial traits, may favor improved survival and adaptation values in changing ecologies ^4^.

**Figure 5.**
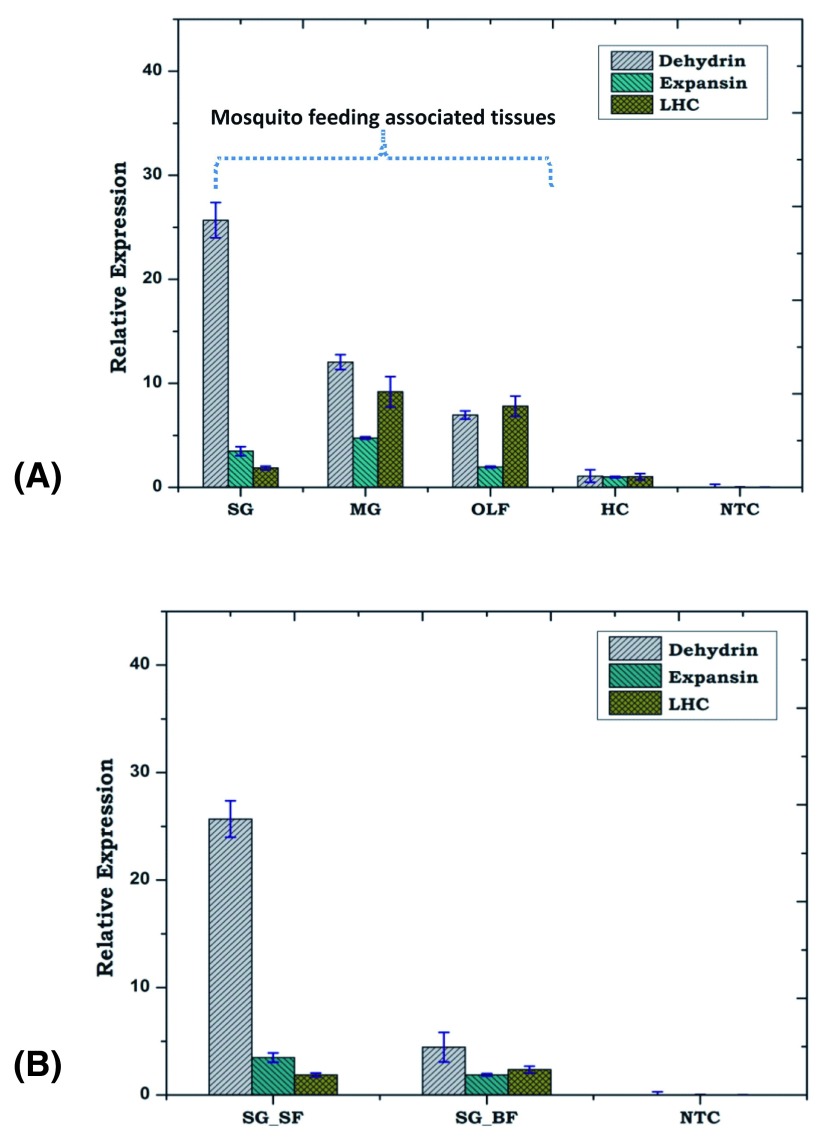
PLTs dominantly express in feeding associated tissues. ( **A**) Tissue specific expression of PLTs; ( **B**) Blood meal response of salivary glands PLTs viz. dehydrin, expansin and light harvesting complex (LHC) in the adult female mosquito. SG_SF: Salivary Gland Sugar Fed; SG_BF: Salivary Gland Blood Fed; MG: Midgut; OLF: Olfactory; HC: Hemocyte; NTC: No Template Control.

With the current available data, including the present investigation, we hypothesize that HGTs in metazoans may also play an important role in the evolution and acquisition of beneficial traits that facilitate feeding and survival adaptation over diverse ecologies. This hypothesis is further strengthened by our following new observations: (a) that PLTs expression seems to be restricted to the tissues, i.e. the feeding machinery components that facilitate digestion and metabolism, e.g. salivary glands, midgut olfactory tissues in case of the mosquito ( Figure 5); (b) absence of PLTs from other non-digestive tissues, e.g. hemocyte (mosquito blood cells); (c) the finding of dominantly associated unique bacterial species to the mosquito digestive tissues viz. salivary gland and midgut ^70^, e.g. *Acidobacteria* (sugar metabolism); *Agromonas* bacteria, a soil oligotroph (nitrogen fixing bacteria) that usually grow at extra low nutrient environments of the paddy field, complementing the high larval density of the mosquito *A. culicifacies* in paddy fields of the rural India ^74^. Indeed, *Agromonas* has been previously isolated from paddy fields ^71^; but largely remain unidentified from any insect species so far.

Furthermore, in mosquitoes it has long been accepted and proven that a significant variation exists in the chromosomal DNA as well as genome size within *Anopheline* and other mosquito species ^75, 76^, but how these variations differentially affect the mosquito biology viz. behavior, physiology, immunity and vectorial capacity etc., are poorly understood at the molecular level.

## Material & methods

**Mosquito rearing:** A cyclic colony of *A. culicifacies* sibling species A, were reared and maintained at 28 ±2°C/RH 80% in the insectary fitted with an automated dawn and dusk simulator allowing a light and dark cycle for 11hrs with 1hr transition from light to dark and vice-versa, essentially required for proper mating and feeding at NIMR ^77^. All protocols for rearing and maintenance of the mosquito culture were approved by the Institutional Animal Ethics Committee (IAEC) of the institute (Reg. No. 33ReBi/GO/S/99/CPCSEA). For our specific research work, pupa stage *A. culicifacies* were collected from the insectary and kept in a round plastic cage fitted with mosquito net, perfectly wiped with 70% ethanol prior to the experiment. Post emergence adult mosquitoes were fed daily on sterile sugar solution (5%) using a glass test tube supplied with a sterile cotton swab throughout the experiment, while mosquitoes were allowed to feed directly on a rabbit for blood meal acquisition. For aquatic development, gravid females were allowed to lay eggs on moistened filter paper mounted inside small plastic cups (e.g. ice cream cup), semi-filled with pre-cooled boiled water. Hatched larvae were feed on mixed dried powder of yeast and fish food (Taiyo Plus, Tetra Gmbh, Germany). The utensils viz. plastic bowl, cup or tray used to rear larvae were properly washed with soap solution, followed by multiple washing with boiled water and air dried. The waste removal and fresh nutrient supply to the larva was maintained with an interval of 24 hrs in 300–400 ml pre-cooled hot water.

### Molecular studies & gene expression analysis

***Tissue collection*:** For the desired tissues viz. salivary glands, midgut, hemocytes, olfactory tissues collection, we followed essentially the same protocol as established for our recent metagenomic and transcriptomic studies ^31, 70^. Briefly, prior to dissection, 3–4 day old sugar or blood fed adult female mosquitoes were surface sterilized using 70% ethanol for 1 min followed by dissection in a sterile water drop on a microscopic slide in a sterile working area under laminar flow. Sterile entomological needles/forceps were used to manually pick up and collect the tissues in the pre-sterilized 1.5 ml Eppendorff tubes containing 50 μl Trizol solution. For the hemocyte collection, a flushing method was opted for as described previously ^80^. Briefly 2–3 μl of Schneider’s (RPMI): FBS: citrate buffer (60:10:30) was injected into the lateral wall of the mesothorax of cold anesthetized mosquitoes, followed by flushing out the diluted hemolymph with an additional 3–5 μl of Schneider’s (RPMI), by clipping of the last abdominal segment. The diluted hemolymph was directly collected by pipette in Trizol. For the egg collection, a clean fine art paint brush was used to scrape the eggs from moistened filter paper, rinsed with sterile water and collected in the Trizol. The other aquatic developmental stages viz. larva, pupae were also manually picked up with a Pasteur pipette, washed with sterile water twice and collected in Trizol for RNA isolation.

***RNA isolation, cDNA preparation and PCR analysis*:** The desired tissues viz. salivary glands, midgut and hemocyte ^78^ or the whole body, were collected in Trizol. Total RNA was isolated using standard Trizol method, followed by first-strand cDNA synthesis using Oligo-dT or Random Hexamer primers (Verso kit). For differential expression analysis, routine RT-PCR and agarose gel electrophoresis protocols were used. Relative gene expression was assessed by QuantiMix SYBR green dye (Biotool Biolabs, Madrid, Spain) in Eco-Real-Time PCR Machine (Illumina). PCR cycle parameters involved an initial denaturation at 95°C for 5 min, 40 cycles of 10 s at 95°C, 15 s at 55°C, and 22 s at 72°C. Fluorescence readings were taken at 72°C after each cycle. A final extension at 72°C for 5 min was completed before deriving a melting curve, to confirm the identity of the PCR product. Actin gene was used as an internal control in all qPCR measurements, where minimum two technical replicates were used in each real-time experiment. To better evaluate the relative expression, each experiment was performed in three independent biological replicates. The relative quantification results were normalized with internal control Actin gene and analyzed by 2 ^–ΔΔCt^ method ^79^.

***PLTs identification and phylogenomic analysis*:** In an attempt to clarify and improve the functional annotation of a cluster of unique sequences encoding plant like proteins, unexpectedly observed from our recently sequenced salivary transcriptomes ^31^, we performed a comparative analysis for both the sugar fed as well as blood fed salivary transcriptomic databases. Initially, to do this we did a species distribution analysis and manually sorted and catalogued the sequences that best match to the plants from the FASTA file. The shortlisted transcripts were subjected to a similarity search against NCBI's NR database using the BLASTx algorithm ^80^, with a cut-off E-value of ≤10 ^−3^ using BLOSUM62 matrix as well as GO annotation/Interproscan analysis using BLAS2GO ^81^. Biocyclic pathway analysis for PLTs KOBAS online ( http://kobas.cbi.pku.edu.cn/home.do) software, version 2.0 ^82^. Following primary BLASTX analysis, the reference sequences from the selected top hits were retrieved and edited for subsequent analysis in the FASTA format. Multiple sequence alignment was performed using ClustaX2, version 2.0 ^83^. The CLC Sequence viewer ( http://www.clcbio.com) software (version 6.9.1) was used for better quality graphics. The phylogenetic relationship was inferred through MEGA5.1 ( http://www.megasoftware.net/) software. The evolutionary history was inferred using the Neighbor-Joining method, and percentage of replicate trees in which the associated taxa clustered together in the bootstrap test (1000 replicates). The evolutionary distances were computed using the p-distance method, presented in the units of the number of amino acid differences per site. A work flow for the Phylogenomic analysis has been presented in the Supplemental material S6. The following major steps were followed:

I. Alignment of orthologous sequences for the selected genes Cysteine Protease, Aquaporin and Alphatubulin using MAFFT v6.864 at default parameters (Auto (FFT-NS-1, FFT-NS-2, FFT-NS-i or L-INS-i) with Amino Acid substitution matrix (BLOSUM62), Gap Penalty (1.53), offset penalty (0.123) and saved in Phylip Interleaved alignment format.
II. Alignment was used to generate RAxML tree, using T-REX online ^84^, at following parameters for generating *de-novo* phylogeny at following parameters: PROTCATDAYHOFF substitution model, Hill Climbing Algorithm, Number of alternative runs on distinct starting trees =100, Rapid bootstrap random seed =12345, Bootstrap random seed =12345. This alternate phylogeny was called H1, as compared to commonly accepted Species phylogeny which was called H0 (the null hypothesis).
III. For Delta SSLS estimation, site wise log likelihood values were calculated using ^85^ for both H0 and H1 phylogeny. Difference in Sitewise Log likelihood was calculated (Delta SSLS= H0-H1), where negative value supports convergent evolution and positive value supports species phylogeny.
IV. For LRT test (Tree Finder), Phylogenetic reconstruction for H0 and H1 was done under WAG substitution model & Likelihood method for identifying best fit protein model with optimized frequencies with Heterogeneity models (G, GI and I). Parametric bootstrapping analysis was done to compare the two evolutionary hypotheses ‘ **H0’ and** ‘ **H1’.** The resulting p-value is the probability that the likelihood ratio simulated under the null hypothesis is less or equal than the observed. Given a level of significance of 5%, a p-value greater than 95% indicates that H1 is better than H0, and a p-value less than 5% indicates that H1 is worse.

***Modeling procedure & 3D structural prediction analysis*:** All structures of representative protein were retrieved from the Protein Data Bank ( www.rcsb.org) and aligned using the structure alignment program STAMP4.0 ^86^. Models using all four structures as templates were generated using Modeller9 version 10 ^87^. 3D representation of the model was prepared in VMD version 1.9 (Visual Molecular Dynamics tool) ^88^.

***Genomic DNA isolation & PCR*:** For the genomic DNA extraction, a total of five adult female mosquitoes, decapitated with head and wing, were collected in extraction buffer and processed as described earlier ^70^. All the PCR amplification conditions and parameters were identical as described above for RT-PCR analysis.

### Immunoblot analysis

**(a)**  **Wheat seedling protein sample preparation:** Wheat seeds were surface sterilized, imbibed for two consecutive days on moist filter pads placed in the glass petridish, under deprived light, given alternate 16h/8h light/dark cycle for 3 days and then processed as described previously ^89^. Briefly, crude protein extract was prepared by homogenization of seeds in phosphate buffered saline (PBS) with added benzamidine hydrochloride (1 mM) and phenylmethylsulfonyl fluoride (PMSF) (1 mM) followed by centrifugation at 15,000 rpm for 30 minutes at 4°C. Supernatant was collected to quantify and optimize the protein sample concentration for SDS-PAGE with different amount of protein (viz. 20 µg, 50 µg, 100 µg, 200 µg and 400 µg). For further experiments 200 µg was selected as an optimal concentration for immunoblot analysis.
**(b)**  **Mosquito developmental stage (egg, larva, pupa) samples:** Different stages of mosquito viz. egg, larva, pupa were collected in PBS containing benzamidine hydrochloride (1 mM) and phenylmethylsulfonyl fluoride (PMSF) (1 mM) protease inhibitors. The collected mosquito whole body samples were homogenized on ice for 10 minutes, followed by centrifugation at 15,000 rpm for 15 min at 4°C. The clean supernatant was collected and quantified for subsequent analysis as described below.
**(c)**  **Bacterial protein sample** : BL21* cells of E. coli (2ml) were grown in LB media containing ampicillin (100 μg/ml) at 37°C till optical density (OD: 600) reached 0.4–0.6. Harvested cells were spinned down at 12000 rpm and re-suspended with 200 µl re-suspension buffer containing 50 mM NaH _2_PO _4_ pH 8.0, 300 mM NaCl, 10 mM Imidazole. Cell lysate was then centrifuged at 12000 rpm for 5 min and clear supernatant was analyzed through SDS-PAGE.
**(d)**  **SDS-PAGE and immunoblot analysis:** Protein samples (200 µg each) were separated on SDS-polyacrylamide gel with Amersham mini vertical electrophoresis system and transferred to nitrocellulose membrane. Membranes were blocked with 1.5% (w/v) gelatin in PBST and incubated with anti-dehydrin primary antibody (affinity purified polyclonal rabbit antiserum; 1:1000 dilution). The unbound antibody was washed three times for 5 min with PBST. Membranes were then incubated with anti-rabbit HRP secondary antibody (monoclonal; 1:60,000 dilution) (Santa Cruz Biotechnology, USA) for 1 hour. Unbound secondary antibody was washed for 5 minutes three times with PBST at room temperature. The blots were visualized using Amersham ECL prime Western blotting detection reagent containing Solution A: luminol enhancer and Solution B: peroxide and developed on X-ray films by developer and bands were readily fixed in fixer solution.
**(e)**  **Immuno-florescence microscopy:** The collected different developmental stages of mosquito viz. egg and pupa were washed with DEPC treated water and fixed with 4% paraformaldehyde (PFA) overnight at 4°C. The PFA was removed with PBST wash followed by dehydration of the samples with a methanol series as described ^90^ and stored at -20°C until use. Before using, the samples were rehydrated with a gradual dilution series of methanol in PBS. Final traces of methanol were removed with PBST washes followed by a final wash with 150 mM Tris HCl, pH 9. Antigen retrieval was proceeded by incubating the samples with 150 mM Tris HCl (pH 9) at 70°C for 15 minutes, which were subsequently permeabilized with chilled acetone at -20°C for 20 minutes. Later blocking was done overnight with 10% BSA in PBST at 4°C. After blocking the mosquito samples were incubated with anti-dehydrin primary antibody (affinity purified polyclonal rabbit antiserum; 1:500 in 1% BSA in PBST) for 3 days. PBST washed samples were incubated with goat anti-rabbit IgG FITC labelled secondary antibody (polyclonal; Santacruz Biotechnology, USA) at 4°C for 2 days. For each washing step with PBST i.e. before permeabilization and/or after primary and secondary antibody incubation, the samples were carefully handled. Finally samples were washed with 4% PFA before mounting with a series of glycerol wash given with 25%, 50% and 75% glycerol in PBS for 20 minutes each. The mounted samples were observed under confocal microscope (Model# A1R, Nikon). Negative control samples were processed in identical conditions, except the use of anti-dehydrin antibody and observed along with test samples.

## Conclusion

Evolution and adaptation of dual feeding (sugar vs. blood) behavior in adult female mosquitoes remains an unresolved question. Comparative salivary transcriptomic and metagenomic analyses provide initial evidence that *A. culicifacies*, may have acquired and evolved with plant like machinery components partly shared by salivary associated microbes, together facilitating feeding preference and adaptation over plants grown in the plain agricultural area of rural India.

## Data availability

The data referenced by this article are under copyright with the following copyright statement: Copyright: © 2015 Sharma P et al.

The sequence data has been submitted to NCBI SRA database under following accession number: AC-SG-SF: SRR1753386. All other data is included as Supplementary material.

## Supplementary material

Supplementary material for ‘Deep sequencing revealed molecular signature of horizontal gene transfer of plant like transcripts in the mosquito *Anopheles culicifacies*: an evolutionary puzzle’.
