# Supplementary material for: Deep sequencing revealed molecular signature of horizontal gene transfer of plant like transcripts in the mosquito Anopheles culicifacies: an evolutionary puzzle
Source: F1000Res. 2015 Dec 30;4:1523. [Version 1] doi: 10.12688/f1000research.7534.1 (PMC4786938; doi:10.12688/f1000research.7534.1)
Supplement: Peer review history

# Peer review: Referee response for version 1

**DOI:** 10.5256/f1000research.8113.r12795

**Published:** 2016-03-10

**Authors:** Luis Boto

**Affiliations:** 1 Department of Biodiversity and Evolutionary Biology(BEB), National Museum of Natural Sciences(MNCN), Spanish National Research Council (MNCN-CSIC)), Madrid, Spain

Sharma *et al*. article presents convincing evidence showing that *Anopheles culicifacies* harbor several plant like genes, probably acquired by horizontal gene transfer, whose expression is associated to the mosquito feeding mode.

Starting from 537 plant like transcripts identified in a previous transcriptomic study, authors present compelling evidence that some of these transcripts are of the mosquito origin (and no contamination) and at least in one case (dehydrin) they encode an expressed and functional protein. In addition, authors discuss the possible role of these acquired plant genes in feeding adaptations.

In my opinion, the article is very interesting and provides a new piece to understand the significance and importance of horizontal gene transfer in metazoan evolution. In this sense, I think that this article deserves to be indexed.

My main concern is in relation to the results presentation (and discussion) through the paper. In this sense, I consider that restructuring the paper and figures could make this interesting paper more readable.

In my opinion, phylogenomic analyses, showing the possible horizontal gene transfer from plants to the mosquito, should precede the presentation of the other results. Identification of mosquito genes related to plant genes is the point that supports and justifies later research and it should be highlighted. In this sense, I suggest a new figure 1 combining the actual Figure 1A and figure 2B and 2C (dehydrin gene tree as an example).

In the same vein, I suggest combining in a new figure 2 the current figure 1B, 1C, 1D, 1E, 1F and figure 5A and 5B. Together, these pieces of information support the mosquito origin of the discussed genes and their involvement in the mosquito feeding mode..

A new figure 3 should show results in dehydrin gene validation encompassing the current figure 2A, 2D, 2E, 2F and 2G.

Other minor points are:

 

1. Authors select a few genes from the initial 537 plant like transcripts pool to demonstrate that some genes are present in the mosquito genome and they are functional. However, many of the initially identified genes are not studied. In this sense, statement in the abstract that “Our multiple experimental validation…..confirmed that plant like transcripts (PLTs) are of mosquito origin” should be softened  to “……..confirmed that **at least** **some of the** plant like transcripts (PLTs) are of the mosquito origin”. In the same sense, a comment in the non-studied PTLs could be welcome through of the manuscript.
2. In the same vein, authors (page 5, “PTLs are of mosquito origin”) say that 10 PTLs are selected for RT-PCR. However in the figure 1B only five of these amplifications are shown. I think that authors should justify why they choose these particular PTLs.
3. On page 8, please review the last sentence in “phylogenomic analysis of plant like transcripts”. It seems that mosquito feeding associated genes acquire new genes.
4. Authors should recognize that Fig.4D is partially modified from Figure 2C in ref.70. In this sense I suggest to change “see another report for details” to “see ref.70 for details” in the Fig. 4 caption.
5. On page 13, authors state that: *The observation of a large pool of chloroplast and nuclear encoded plant genes in the mosquito transcriptome supported the previous finding of similar gene transfer of photosynthetic machinery components from algae to mollusk. *As the authors recognize in another place in the article, transfer of algal genes to *Elysia chlorotica* is controversial and in any case, authors should be cautious in the use of their results to support an absolutely different case study. In this sense I also suggest to soften claims for support to other similar observations in the Abstract.

I have read this submission. I believe that I have an appropriate level of expertise to confirm that it is of an acceptable scientific standard.

# Peer review: Referee response for version 1

**DOI:** 10.5256/f1000research.8113.r12793

**Published:** 2016-03-09

**Authors:** Vas Dev

**Affiliations:** 1 National Institute of Malaria Research (NIMR Field Unit), Indian Council of Medical Research(ICMR), Guwahati, Assam, India

The article by Sharma and group, is one of the first reports on mosquito-microbe-plant interaction, defining the molecular relations of how mosquitoes might have evolved from herbivores to blood feeders. In fact this is a long standing question in the insect communities, where this report supports the idea that mosquitoes may have acquired the plant genes for specific functions. Although the functions of the genes are yet to be established, observation of photosynthetic genes and microbes, appears to facilitate the photo-light meditated responses, probably in feeding and/or mating. To the best of my knowledge and experience the behavior of this mosquito species ( *Anopheles culicifacies*) is quite complex compared to other malarial vector species.

The most interesting part is that the homology search analysis of PLTs reveals the name of many local plant species which commonly grown in plain areas of rural India. I believe these observations will serve as a basis for new leads for authors to understand how mosquito-plant interactions facilitated this mosquito species to evolve to feed and adapt in plain agricultural areas.

I have read this submission. I believe that I have an appropriate level of expertise to confirm that it is of an acceptable scientific standard.
